# Embedding brief interventions for alcohol in general practice: a study protocol for the REACH Project feasibility trial

**DOI:** 10.3399/BJGPO.2021.0037

**Published:** 2021-06-02

**Authors:** Elizabeth Sturgiss, Nilakshi Gunatillaka, Lauren Ball, Tina Lam, Suzanne Nielsen, Renee O'Donnell, Chris Barton, Helen Skouteris, Chun Wah Michael Tam, David Jacka, Danielle Mazza, Grant Russell

**Affiliations:** 1 Department of General Practice, School of Primary and Allied Health Care, Monash University, Melbourne, Victoria, Australia; 2 Menzies Health Institute Queensland and School of Allied Health Sciences, Griffith University, Brisbane, Queensland, Australia; 3 Monash Addiction Research Centre, Eastern Health Clinical School, Monash University, Frankston, Victoria, Australia; 4 Health and Social Care Unit, NHMRC CRE in Health in Preconception and Pregnancy (CRE HiPP), School of Public Health and Preventative Medicine, Faculty of Medicine, Nursing and Health Sciences, Monash University, Melbourne, Victoria, Australia; 5 Warwick Business School, The University of Warwick, Coventry, UK; 6 Primary and Integrated Care Unit, South Western Sydney Local Health District, Liverpool, New South Wales, Australia; 7 School of Population Health, Faculty of Medicine, University of New South Wales (UNSW), Sydney, New South Wales, Australia; 8 Monash Health, Melbourne, Victoria, Australia

**Keywords:** primary health care, general practice, alcohol use disorder, low income population, feasibility studies

## Abstract

**Background:**

Alcohol is a major source of harm in Australia that disproportionately affects low-income communities. Alcohol brief interventions (ABIs) combine an assessment of a person’s alcohol use with advice to reduce health risks. Despite their effectiveness, ABIs are not routinely performed by clinicians. This article presents a protocol for a feasibility trial of pragmatic implementation strategies and a new set of resources to support clinicians to complete ABIs in Australian general practices.

**Aim:**

To explore the facilitators and barriers to increasing the uptake of ABIs in primary care, including acceptability, reach, adoption, fidelity, and sustainability.

**Design & setting:**

A mixed-methods evaluation of the uptake of ABIs in general practice clinics serving low-income communities in Melbourne, Australia. The approach is informed by the Consolidated Framework for Implementation Research (CFIR) and Normalisation Process Theory (NPT).

**Method:**

The implementation strategies and resources will be trialled in five general practices over 12 months. The primary outcome will be change in the proportion of adult patients with a complete alcohol history in their electronic medical records. Baseline data collection includes a practice survey to describe practice routines for ABIs and de-identified patient medical record data on completed alcohol histories (repeated at 3, 6, 9, and 12-months post-intervention). Survey and interview data will also be collected from clinicians, patients, and primary health network staff to assess acceptability and feasibility of the intervention.

**Conclusion:**

The study will explore how the implementation strategies and resources can improve alcohol screening and management among low-income patients in general practice.

## How this fits in

Brief interventions for alcohol delivered in primary care are effective for reducing alcohol-related harm. Currently, ABIs are often not routinely delivered in primary care. A new implementation strategy has been developed with supporting resources to increase the uptake of ABIs in primary care. This implementation trial will explore barriers and facilitators to increasing routine delivery of ABIs in primary care, to inform future policy and practice.

## Introduction

### Background and rationale

Alcohol is a major source of harm. Each year, harmful alcohol use contributes to 3 million deaths and the loss of 132.6 million disability-adjusted life years (DALYs).^1^ Low-income communities are at increased risk of alcohol-related harm.^2^ In Victoria, Australia, the burden of the 1200 deaths per year attributable to alcohol^3^ falls disproportionately on the 13% of Victorians living in poverty.^4^

ABIs involve assessing the amount of alcohol a person is consuming and offering individualised advice to reduce the associated health risks.^5^ ABIs provided by GPs and nurses in community-based primary care can reduce the number of episodes of risky drinking and weekly average alcohol consumption among people with problematic alcohol use.^5^ Alcohol-related harms affect more people with harmful alcohol use than those with alcohol dependency, and large reductions in alcohol-related harms can be achieved by reducing alcohol use in the former population.^6,7^

Strong primary care systems are the foundation of equitable healthcare service delivery.^8^ In the setting of alcohol harm, equity is especially important as people from low socioeconomic groups experience a disproportionate amount of harm from alcohol use.^9^ Few trials on the effectiveness of ABIs have considered the specific needs of low-income groups, potentially contributing to greater health disparities.^10^ The overall aim of the project is to: (a) increase screening for problematic alcohol use; and (b) increase the application of ABIs in general practice. The preferential focus on low-income groups will aim to reduce health inequity by ensuring the approach is most acceptable, feasible, and effective for low-income groups.^11^

### Objectives

Mixed methods will be used to assess the acceptability and feasibility of an implementation strategy to increase the uptake of ABIs for alcohol in Australian general practices serving low-income communities.

## Method

This is a single-arm implementation trial using mixed methods to evaluate the uptake of ABIs in primary care. The approach is informed by: 1) the CFIR to assess factors affecting implementation and effectiveness; and 2) NPT to understand how change is embedded in a practice.

### Study setting

The trial will be conducted in five general practice clinics located in northern metropolitan Melbourne, Australia, in a region corresponding to a primary health network (PHN) catchment (PHNs are federally funded to oversee primary care delivery in local regions).^12^

Participating general practice clinics will be located in a low-income area as identified by the PHN; that is, a Socio-Economic Indexes for Areas (SEIFA) score <1000, where a score of 1000 is the mean for all areas, and scores lower than this indicate relative disadvantage. Practices will use electronic patient medical record and billing software compatible with the PHN’s practice data extraction tool (Pen CS CAT4).^13^ Consent will be gained from practice management and at least one GP. No specific eligibility criteria will apply to participating GPs. No clinician in the practice will be mandated to use the resources. Patient participants will be aged >18 years and able to understand eighth-grade English. Interpreters will be used for patient interviews when necessary.

### Intervention

In preparation for this trial, the authors sought to understand participants’ experiences of talking about alcohol in general practice settings and suggestions on how to promote and improve these conversations (Sturgiss E, Lam T, Russell G, *et al*. *Patient and clinician perspectives of factors that influence the delivery of alcohol brief interventions in Australian primary care: A qualitative descriptive study —* under review). Implementation strategies and an associated resource pack to increase the uptake of ABIs in primary care were then co-designed with patients and clinicians (https://www.monash.edu/medicine/spahc/general-practice/research-projects/reach).

NPT^14^ was used as well as 'priming' to construct the approach to implementation. The authors incorporated 'sense making' for clinicians with training and resources on best practices for ABIs, 'relational work' by identifying and supporting practice champions, 'operational work' using in-consultation resources, 'appraisal work' with regular updates to the clinics on alcohol screening rates, and 'priming' the patients with posters and pamphlets to be more receptive to discussions about alcohol. Before the start of the trial, a state of emergency was declared in Australia owing to the COVID-19 pandemic. The intervention was then adapted for use during telehealth consultations (see Supplementary Table S1).

### Ethical issues

Ethics approval has been granted (see statement below). Routine systems are in place to offer assistance and follow-up any patient who is distressed by health-related research. This includes signposting opportunities for support plus personal follow-up at participant request. Survey and interview participants who express concerns about their alcohol use will be directed to seek help from their primary care provider or local drug and alcohol counselling services.

### Intervention process

Before the trial, practices will identify a champion to promote the intervention to their colleagues. Practice engagement staff from the PHN will support implementation at the practice with quarterly visits to provide ongoing feedback on practice performance and to promote use of the clinical resources. This rollout via the PHN was chosen as a pathway to support sustainability for future scale-up.

### Outcomes

The primary outcome is the change in the proportion of adult patients (aged >15 years) with completed alcohol histories in their electronic medical record. Implementation outcomes, informed by RE-AIM (Reach, Effectiveness, Adoption, Implementation, and Maintenance),^15^ include:

- Reach: the change in proportion of patient records with information on alcohol status (drinks alcohol; does not drink alcohol) as a proxy marker for where a brief intervention (BI) is likely to have occurred.
- Acceptability: to patients, clinicians, practice staff, and PHN staff.
- Adoption within each practice and within the PHN processes.
- Fidelity of intervention implementation via project timelines completed by the PHN, research team, and member-checked during provider interviews.
- Sustainability as perceived by practice staff, clinicians, and PHN staff.

### Sample size

Five practices will be recruited to evaluate the process of implementation. At least one GP or practice nurse will be formally recruited at each practice; other clinicians will have access to the resources and can participate in team feedback meetings. For the nested short message service (SMS) survey study, 140 patients will be recruited who self-identify as drinking at risky levels across the five practices per the AUDIT-C questionnaire.^16^ The authors are interested in the response rate over time; that is, both the number of responses at each time point, and the number of questions answered at each time point. Changes will also be able to be detected in drinking patterns (10% change total standard drinks per week compared with baseline, power 0.8, significance 0.05).

### Recruitment

#### Practices

The PHN will recruit practices via newsletters and the PHN website. A member of the research team will then contact practice management to explain the project and seek written informed consent.

#### Practice staff

Researchers will ask practice management to circulate information to their clinicians. Interested clinicians will contact the researchers for more information and to provide written consent.

#### Patients

The practice will send an SMS to all patients who visited the practice in the previous 3 months, inviting them to fill in a short survey on their experiences of discussing alcohol with their clinician. Patients will also indicate if they are happy to be contacted for a follow-up survey and/or interview. Follow-up surveys will be sent to participants who self-identify as drinking at risky levels.

### Data collection methods

Table 1 shows data collection at the patient, provider, practice, and PHN level.

**Table 1.** Data Source and data collection timepoints

Data source	Practice	Provider	Patient	Primary health network
Tool or survey	*Electronic patient database (PENCS CAT4^TM^*^13^)	Practice survey	NoMAD tool^a^	Interview	Patient survey (all)	Interview	Follow-up SMS survey	Interview	Project timelines
Contact point timeline									
Baseline	X	X							
Post- consultation					SMS link	Telephone			
3 months	X						X	X	X
6 months	X		X				X	X	X
12 months	X		X	X			X		X

SMS = short message service.

a Normalisation Measure Development questionnaire.^17^

### Quantitative instruments

1. *De-identified patient data from Pen CS CAT4.*^13^ The PHN receives data from all practices in the catchment relating to completion of patient alcohol histories. A data-sharing agreement will be entered into with the PHN and participating practices to access this data. The PHN will provide data on the patients with complete alcohol histories as determined by recording of 'drinker', 'non-drinker', 'nothing recorded', and 'patient <15 years of age with nothing recorded' in the patient’s electronic medical record. The baseline measure will include all active patients; that is, those with at least three visits in the past 2 years. At 3 months and 6 months, the change in proportion of patients with an alcohol history will be measured by comparing the proportion of patients with at least one visit to the practice in the preceding 3 months who had a complete alcohol history. These measures will be available for all other clinics (approximately 850) in the same catchment area for comparison.
2. *Practice survey*. This will be administered at baseline to collect information about the practice’s structure, staffing, record management systems, patient load and demographics, and processes for patient intake and assessment relevant to ABIs.
3. *NoMAD* (Normalisation Measure Development) *tool.^17^* The NoMAD tool is a quantitative survey to be completed by participating GPs, practice nurses, and practice administrative staff to assess how well ABIs were embedded into everyday practice.
4. *Patient survey*. This will be via SMS to capture data on whether patients were asked about alcohol use, how they found the experience, their alcohol use (AUDIT-C),^16^ and demographics including low income status.
5. *Patient survey for patients with risky alcohol use*. Patients who self-report risky alcohol use (AUDIT-C) in the patient survey will be invited to participate in quarterly follow-up SMS surveys. Information will be collected on:

- The average weekly consumption of alcohol in standard drinks.
- The frequency of episodes of high-risk drinking.
- The number of attendances at the general practice.

A subgroup analysis based on self-reported low-income status will be completed.

#### Qualitative instruments

The CFIR interview guides will inform the interviews tailored for each participant group.^18^

1. *Patient interview*. Twenty patients will be interviewed to provide further feedback about the acceptability of the intervention. Patients will be purposively sampled with self-reported low-income, and maximum variation will be considered in sex, age, and self-reported consultation experience when inviting patients for an interview. Interviews will focus on the acceptability of the intervention for patients, suggested improvements, and any unintended consequences.
2. *Clinician interview*. At least two clinicians from each practice will be interviewed to assess how the intervention works within the consultation and any unexpected effects. Interviews will focus on the feasibility of the intervention in daily practice, suggested improvements, and sustainability of the intervention.
3. *PHN interview*. Up to five PHN staff who have been involved in the implementation of the REACH resources will be interviewed. The research team will focus on The CFIR 'outer setting' to better understand how the broader policy environment has influenced the implementation process.

### Data analysis

Both qualitative and quantitative data will be used to assess the acceptability, feasibility, and relative effectiveness of the intervention. The data will be collected concurrently and integrated to gain a better understanding of the implementation process for REACH.

#### Quantitative analysis

Quantitative data will be analysed descriptively, with means and standard deviations or medians and ranges reported for continuous variables, and proportions for categorical variables. Correlations will be calculated using Spearman rho (ρ) owing to the anticipated non-normal distribution of scores. Repeated measures data will be analysed using a non-parametric statistics such as the McNemar and Wilcoxon signed-rank test. Multiple regressions will be conducted to assess associations between the intervention measures.

#### Interrupted time series

An interrupted time series analysis will be performed using data from the enrolled clinics, as well as the 850 clinics within the same catchment to determine how much alcohol screening has changed in the intervention clinics and across whole PHN over the study period.

Analyses will be computed in SPSS (version 24).

#### Qualitative analysis

Audio files of interviews will be de-identified and professionally transcribed. Analyses will be conducted using NVivo (version 10 or higher).

Although the interview guides will be based on CFIR, inductive thematic coding will be used to ensure the findings are grounded in the data and not a pre-existing framework.

Summarised findings and early interpretations will be discussed with the research team in regular small team meetings. The authors will also meet on a minimum of two occasions with the entire investigator team to finalise the themes from the data.

Table 2 outlines the approach to the mixed-methods analysis of the data.

**Table 2.** Mixed-methods data collection, analysis, and outcomes: implementation matrix adapted from Guetterman *et al* ^19^

**Data type**	**Study aim**	**Data collection procedure**	**Data analysis** **procedure**	**Theoretical framework**	**Products or outcomes**	**Points of integration**
Qualitative	To identify the barriers and facilitators to implementing alcohol BIs in general practice	Interviews: patients; clinicians; practice staff; PHN staff	Inductive thematic coding	CFIRRE-AIM	Perception of implementation processes from multiple viewpoints (acceptability; adoption; fidelity)	Triangulate with NoMAD data to inform implementation process.Compare with practice level data on % alcohol intake recording to look for patterns on increased uptake, or not
Quantitative	Increase uptake of alcohol BIs	Routine data extraction from practice to PHN (drinker, non-drinker, not recorded; sex; age); PHN to share the amalgamated data with research team	Descriptive statistics every 3 months.Interrupted time-series analysis compared with all practices in PHN catchment. Data will be collected monthly	CFIRRE-AIM	% change in patient records with alcohol intake recorded (reach; adoption)	Compare with qualitative interview data to understand barriers and facilitators to % change
Quantitative	To identify the barriers and facilitators to implementing alcohol BIs in general practice	NoMAD survey from practice managers and clinicians	Likert scale	Normalisation Process Theory	Measure of provider assessment of potential 'normalisation' of new procedure (acceptability; adoption; sustainability)	Triangulate with interview data from providers to identify implementation processes
Quantitative	Nested SMS study to determine response rates to SMS surveys over 9–12 months	SMS survey to patients at 3-monthly intervals using 2-way SMS (Qualtrics)	% response rate% of questions completed at each time point	NA	Response rate to SMS surveys over time	NA

BI = brief intervention. CFIR = Consolidated Framework for Implementation Research. NA = not applicable. NoMAD = Normalisation Measure Development. PHN = primary health network. RE-AIM = Reach, Effectiveness, Adoption, Implementation, Maintenance. SMS = short message service.

## Discussion

The implementation trial will generate evidence on the effectiveness of the implementation strategy at increasing the uptake of ABIs in primary care, as well as the acceptability and feasibility of this strategy, with a particular focus on low-income patients. Implementation and behaviour change theory have been used to guide both the design of ABI strategy and resources, and the approach to the evaluation. The co-design approach and existing collaborations are strengths of this work. The work will be influenced and shaped by the global pandemic. The unique opportunity will be used to learn more about primary care delivery in high-risk situations that may be useful in other disaster settings (for example, bushfire, or flood). There is potential that the pandemic may alter the implementation findings and that some will be inapplicable to non-disaster settings. New knowledge will be generated on how similar interventions can be adapted for telehealth consultations and how preventive health care is affected by a global pandemic.
